# Large Bowel Obstruction Due to a Big Gallstone Successfully Treated with Endoscopic Mechanical Lithotripsy

**DOI:** 10.1155/2015/798746

**Published:** 2015-06-02

**Authors:** Marco Balzarini, Laura Broglia, Giovanni Comi, Calcedonio Calcara

**Affiliations:** Gastroenterology Unit, ASL Novara, 28021 Borgomanero, Italy

## Abstract

Colonic gallstone ileus in an uncommon mechanical bowel obstruction caused by intraluminal impaction of one or more gallstones. The surgical management of gallstone ileus is complex and is potentially of high risk. There have been reports of gallstone extractions using various endoscopic modalities to relieve the obstruction. In this report we present the technique employed to successfully perform a mechanical lithotripsy and extraction of a large gallstone embedded in a sigmoid colon affected by diverticular stenosis. We passed through the stenosis with a 11.3 mm videoscope with 3.7 mm channel. A large lithotripsy extraction basket was used to catch and break up the stone and fragments were removed using the same basket. The patient was discharged asymptomatic three days after the procedure. Using appropriate devices mechanical lithotripsy is a safe and effective method to treat colonic obstruction and avoid surgery in the setting of gallstone ileus even in case of big stones.

## 1. Introduction

Despite gallstone ileus accounting for 25% of mechanical small bowel obstruction in patients over 65 years of age, colonic gallstone obstruction is a rare event and surgical intervention is usually required [1]. Although this has been shown to be successful, different endoscopic options to remove gallstone from the large bowel were attempted including extracorporeal lithotripsy, Dormia baskets, and polypectomy snares [2–5]. Usually these techniques were considered to be successful in situations where the residual lumen is big enough to allow the passage of the colonoscope and the gallstone is small enough to be endoscopically extracted. We present the case of an elderly woman with a large calcified stone impacted in a stricture of the sigmoid colon who was successfully treated with one session of endoscopic mechanical lithotripsy.

## 2. Case Report

**Figure 1:**
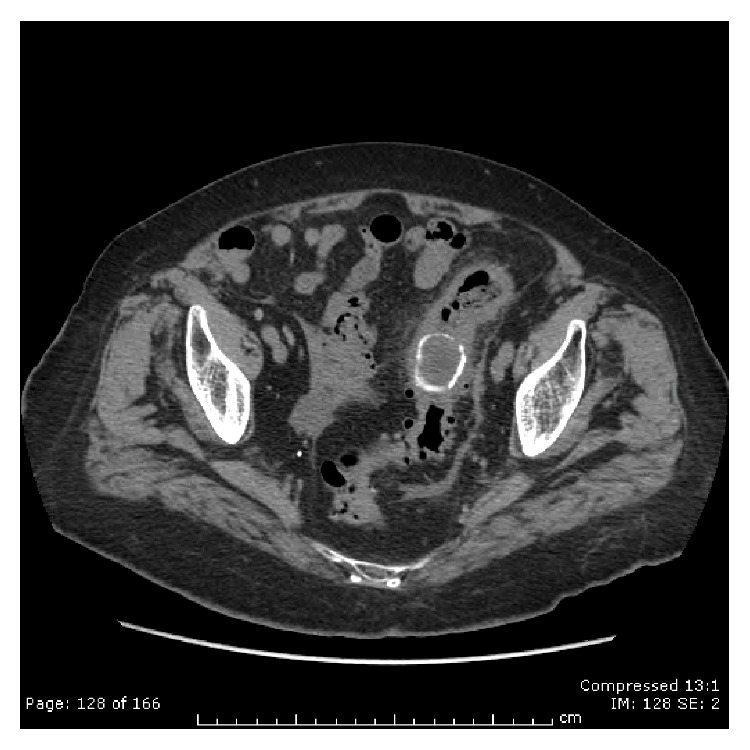
Computed tomography. Gallstone impacted in the sigmoid colon.

**Figure 2:**
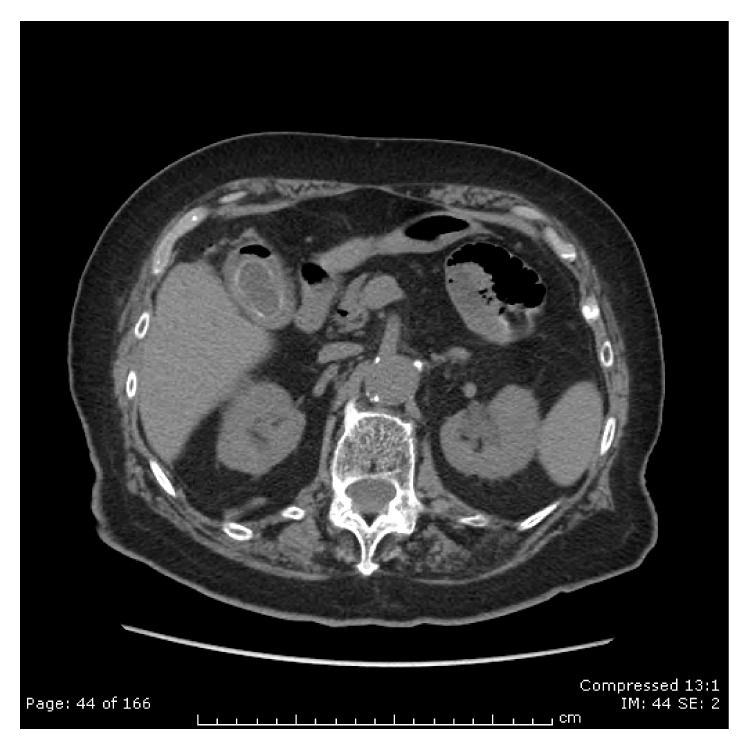
Computed tomography. Pneumobilia.

**Figure 3:**
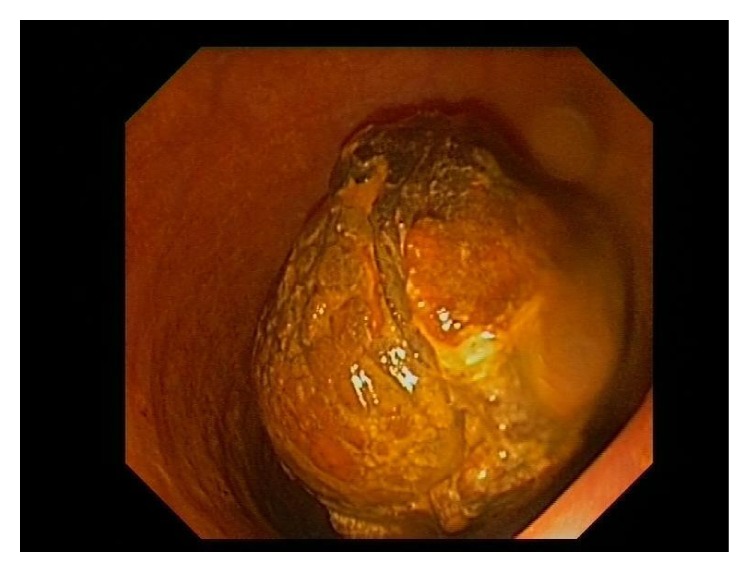
Endoscopy image big gallstone.

**Figure 4:**
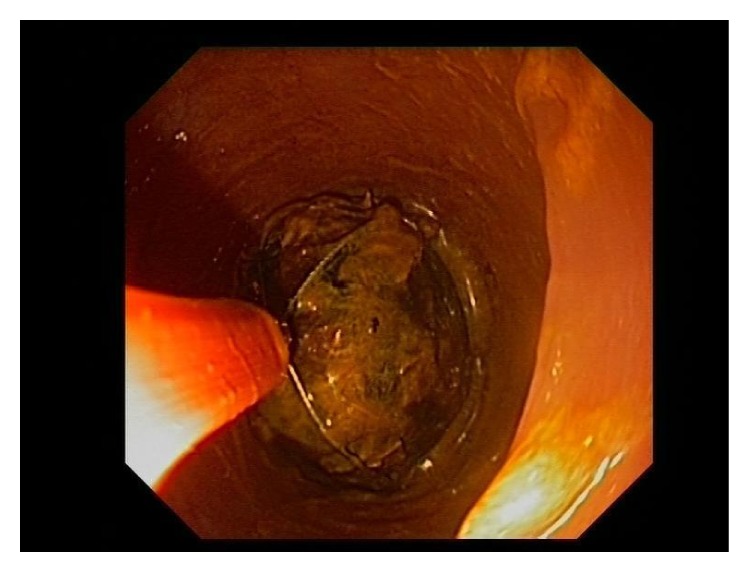
Gallstone into lithotripsy extraction basket.

A 94-year-old female was admitted to the hospital with a 5-day history of obstipation associated with vomiting and abdominal pain. On examination the vital signs were stable, and her abdomen was mildly distended but no tender and no palpable masses or hernia was detected. The rectal examination was also normal. The only symptoms evident prior to the operation were for headaches and dizziness; she never had abdominal surgery in the past. The plain abdominal X-ray revealed the presence of a round calcified image in the left pelvis. Computed tomography demonstrated extensive sigmoid diverticulosis and the presence of a 3,5 cm stone impacted in the sigmoid colon (Figure 1), in addition to a separate 3 cm stone within the gallbladder lumen. There was fistulous communication between the gallbladder and the colon with resultant pneumobilia (Figure 2). Due to her advanced age the patient was unfit for major surgery (i.e., laparotomy and enterotomy to remove the stone) and endoscopic intervention was recommended. The patient provided informed written consent prior to the procedure. In the endoscopy suite, under conscious sedation (Midazolam 2 mg), the patient was placed in the supine position. An Olympus videoscope (GIF 1TQ160 Olympus) with 11.3 mm body and a 3.7 channel was introduced transanally. We used CO_2_ insufflation during the procedure. The stone was found to be embedded above a 15 cm long area of narrowing, likely from prior diverticulitis (Figure 3). A 3 cm × 6 cm 10 French lithotripsy extraction basket (FS-LXB-3X6 Cook Medical) was passed through the biopsy channel of the videoscope. Due to the large basket size it was possible to catch the stone and perform lithotripsy (Figure 4). Smaller stone fragments were removed using the same basket and the obstruction was relieved. During hospital stay the patient was fed intravenously until recovery. The patient was not considered a candidate for further surgical or endoscopic treatment and recovered without any complications related to the endoscopic procedures. She was discharged asymptomatic on postoperative day 4.

## 3. Discussion

Large bowel obstruction due to impacted gallstone is uncommon and only few reports in the literature are present. Although spontaneous resolution of gallstone ileus is described [6], it generally causes acute bowel obstruction, and the aim of the treatment is gallstone extraction. Surgical enterolithotomy is the traditional treatment and this allows for relief of the obstruction in the short term. Fistula resection can be safely performed during a second surgical procedure if indicated [7]. As this condition is predominantly encountered in an elderly population with a high incidence of comorbid conditions, the complication and mortality rates of surgical treatment are substantial. Endoscopically accessible impacted gallstones are amenable to less invasive alternative therapeutic options including intracorporal laser lithotripsy and endoscopic mechanical lithotripsy for fragmentation but usually it is not possible to use Dormia basket in case of big stones while intracorporal laser lithotripsy is not commonly available. Ultrasound-guided ESWL has also been suggested as a noninvasive alternative to surgery to fragment the stone and solve the occlusion [8] in case of failure endoscopic removal. In our case report the possibility to access the obstruction endoscopically suggested a conservative treatment approach. In the setting of diverticular disease and colonic occlusion we used CO_2_ insufflation to minimize the risk of perforation. Despite the large size we successfully removed a gallstone embedded in a sigmoid stricture using mechanical lithotripsy. This was possible combining the use of a slim videoendoscope with a large lithotripsy basket. This technique and other endoscopic approaches should be regarded as an effective and safer alternative to surgery.

## Core Tip

Gallstone ileus is predominantly encountered in an elderly population with high complication and mortality rates related to surgical treatment. We describe the use of an endoscope with large channel and slim body combined with a large lithotripsy extraction basket to remove a large gallstone embedded in a sigmoid colon affected by diverticular stenosis. No surgery was performed, and the patient was discharged 3 days after the procedure.
